# Targeting the Tumor Immune Microenvironment in Triple-Negative Breast Cancer: The Promise of Polyphenols

**DOI:** 10.3390/cancers17172794

**Published:** 2025-08-27

**Authors:** Aaron L. Hilliard, Tanya D. Russell, Patricia Mendonca, Karam F. A. Soliman

**Affiliations:** 1 Division of Pharmaceutical Sciences, College of Pharmacy and Pharmaceutical Sciences, Institute of Public Health, Florida A&M University, Tallahassee, FL 32307, USA; patricia.mendonca@famu.edu (P.M.); karam.soliman@famu.edu (K.F.A.S.); 2 Center for Advancing Professional Excellence, University of Colorado Anschutz Medical Campus, Aurora, CO 80045, USA; tanya.russell@cuanschutz.edu; 3 Department of Biology, College of Science and Technology, Florida A&M University, Tallahassee, FL 32307, USA

**Keywords:** triple-negative breast cancer, tumor immune microenvironment, CCL2, PD-L1, polyphenols, natural compounds

## Abstract

Breast cancer remains a formidable global health challenge, with triple-negative breast cancer (TNBC) posing unique clinical complexities. Characterized by its aggressive nature and limited number of specific therapeutic targets, this breast cancer subtype disproportionately affects African American women, highlighting critical disparities in care. The tumor immune microenvironment (TIME) plays a critical role in breast cancer development and response to immunotherapy, and it is essential in fostering an immunosuppressive and pro-inflammatory niche. Inflammation, primarily mediated by the NF-κB signaling pathway and chemokine signaling, particularly involving CCL2, plays a pivotal role in TNBC progression and therapy resistance. This review describes some of the molecular mechanisms of polyphenols, which are naturally occurring compounds abundant in various dietary sources, and their potential use as therapeutic agents in the management of TNBC. Polyphenolic compounds have been described as modulating the TIME through the inhibition of tumor progression, immune evasion, and therapy resistance, due to their diverse bioactivities, including anti-inflammatory, antioxidant, and anticancer properties, making them attractive candidates for combating the aggressiveness of TNBC and addressing treatment disparities. Polyphenols, such as curcumin, gossypol, butein, epigallocatechin gallate, cardamonin, and resveratrol, have demonstrated efficacy in modulating several signaling pathways within the TIME, which are implicated in the progression of TNBC. This review highlights the potential effects of polyphenols on inflammatory cytokine release, programmed cell death ligand 1 (PD-L1) expression, which is associated with immune evasion by the host cell, and various intracellular signaling cascades, demonstrating their potential use in personalized therapeutic interventions for TNBC. This study also describes differential responses of TNBC cell lines to polyphenol treatment, highlighting the importance of considering genetic variability in therapeutic strategies, as well as the importance of the interaction of polyphenols with the gut microbiome, which may establish the bioavailability and effectiveness of these compounds toward therapeutic outcomes. Further preclinical and clinical studies are warranted to fully elucidate the therapeutic potential of polyphenols and translate these findings into clinical practice, thereby improving outcomes for patients with TNBC worldwide.

## Simple Summary

Triple-negative breast cancer (TNBC) is an aggressive form of breast cancer that lacks targeted treatment options and disproportionately affects African American women. The tumor immune microenvironment (TIME) plays a key role in promoting tumor growth, immune evasion, and therapy resistance. This review explores how polyphenolic natural compounds found in foods like turmeric, green tea, grapes, and berries can influence the TIME in TNBC. Polyphenols such as curcumin, resveratrol, gossypol, cardamonin, butein, quercetin, and epigallocatechin gallate have shown promise in reducing inflammation, blocking tumor-promoting signals, and enhancing immune responses. By targeting TIME and understanding how the gut microbiome affects polyphenols bioavailability may offer safer and more effective adjuvant treatment strategies for patients with TNBC.

## 1. Introduction

Breast cancer remains a significant global health concern, posing substantial challenges to healthcare systems worldwide. In 2022 alone, an estimated 2.3 million new cases emerged, underscoring its status as the most commonly diagnosed cancer among women and the primary cause of cancer mortality [1]. This malignancy not only ranks as the most commonly diagnosed cancer but also stands as the foremost cause of cancer mortality, emphasizing the urgent need for comprehensive approaches to its prevention and management [1]. On a national scale, the American Cancer Society’s 2024 estimates projected approximately 310,720 new breast cancer cases in the United States, with approximately 42,250 related deaths [2]. Despite a lower overall incidence of breast cancer, African American women experience disproportionately higher mortality rates compared to other racial and ethnic groups. Specifically, other ethnicities’ lifetime probability of developing breast cancer stands at 11.6% (1 in 9), compared to 13.6% (1 in 7) for African American women [3]. Furthermore, the lifetime probability of dying from breast cancer is higher at 3.0% (1 in 33) for African American women compared to 2.5% (1 in 39) for the general population [3].

African American women with breast cancer also face challenges related to late-stage diagnosis, which leads to poorer prognoses, more extensive treatments, and reduced treatment success rates [3]. These disparities are particularly evident in the context of triple-negative breast cancer (TNBC), a subtype characterized by the absence of estrogen receptor (ER), progesterone receptor (PR), and human epidermal growth factor receptor 2 (HER2). TNBC accounts for 10–15% of all breast cancer diagnoses and presents unique therapeutic challenges due to its lack of responsiveness to standard hormonal and HER2-targeted therapies [4,5]. Of particular concern is the disproportionate burden of TNBC among African American women, who are twice as likely as women of other racial and ethnic groups to be diagnosed with this aggressive subtype [3,6]. Additionally, TNBC incidence rates are approximately twice as high in Black women compared to their White counterparts [6]. This disparity underscores the urgent need to address TNBC, particularly among underserved populations, through targeted interventions and therapies.

Most of the TNBC treatments consist of chemotherapy, which can reduce the tumor size in the beginning, but its overall efficiency is limited, with many side effects during the treatment. Also, there is a failure to entirely prevent recurrence, which may lead to drug resistance, reducing the success of a long-term treatment [7]. The prominent TNBC heterogeneity and aggressiveness highlight the need to develop different approaches that can overcome these burdens with minimal toxicity to patients [8].

In the last few decades, natural compounds have been studied because of their ability to target several signaling pathways associated with cancer development, offering less toxicity [9]. Among this group, flavonoids represent a diverse class of polyphenolic substances commonly found in medicinal plants, fruits, and vegetables. Flavonoids have shown antioxidative, anti-inflammatory, and anti-cancer activities [10], working through multiple pathways that influence cell growth, apoptosis, and cancer cells’ invasion [9]. These polyphenolic compounds are vital because they can cause cancer cells’ death, while normal cells remain relatively unaffected, thus improving their suitability in cancer therapy [11].

Polyphenolic compounds have been shown to affect tumor progression, immune evasion, and therapy resistance by modulating the tumor immune microenvironment (TIME), which is essential in fostering an immunosuppressive and pro-inflammatory niche. The TIME mainly consists of tumor cells, vasculature, and extracellular matrix, which influence tumor initiation and therapy response [12]. The dynamic nature of the TIME enables tumor cells to suppress immune responses through the secretion of cytokines, growth factors, and tissue matrix remodeling [13]. Understanding the TIME within TNBC enhances the ability to predict and guide immunotherapeutic responses while uncovering new therapeutic targets. Given the potential of polyphenols to influence immune responses, it is of great interest to study their biological impact, specifically on TNBC-derived TIME.

Given the urgent need to address the challenges posed by TNBC, particularly among underserved populations like African American women, there is a growing interest in exploring novel therapeutic approaches. Targeting the TIME components through NF-κB activation, transcription of inflammatory mediators, and modulation of the programmed cell death 1/programmed cell death ligand 1 (PD-1/PD-L1) signaling represents a promising therapeutic strategy for combating the aggressiveness and drug resistance observed in TNBC treatments. Considering these challenges, exploring novel therapeutic avenues, such as the potential role of natural compounds like polyphenols, holds promise in advancing TNBC research and addressing the unique needs of underserved populations. Plant-derived polyphenols have emerged as promising candidates owing to their anti-inflammatory, antioxidant, and anticancer properties [14,15]. Understanding the complexities of TNBC and harnessing the therapeutic potential of polyphenols represent crucial steps in improving outcomes and reducing disparities in breast cancer care. Therefore, this review focuses on the possibility of several polyphenols modulating the TIME as a therapeutic approach to enhance TNBC patients’ outcomes and decrease therapy resistance.

## 2. The TIME

TIME plays a central role in the progression, metastasis, and therapeutic resistance of TNBC. It is a dynamic and complex ecosystem composed of tumor cells, immune cells (e.g., T lymphocytes, macrophages, natural killer cells), stromal cells, vasculature, and extracellular matrix components [12]. TIME fosters an immunosuppressive milieu that promotes tumor growth and hinders effective immune surveillance and therapeutic efficacy. Chronic inflammation is a hallmark of TIME in TNBC and is primarily driven by signaling cascades such as the nuclear factor kappa B (NF-κB) pathway and pro-inflammatory chemokines such as the chemokine (C-C motif) ligand 2 (CCL2), which recruit tumor-associated macrophages (TAMS) and other immune-suppressive cell types [16]. The presence of immune checkpoint molecules such as programmed cell death protein 1 (PD-1) and its ligand (PD-L1) further enables tumor cells to evade immune detection and destruction. Understanding the structure and function of the TIME is critical to advancing immunotherapeutic approaches in TNBC. Emerging evidence indicates that targeting components of the TIME, including inflammatory mediators, immune checkpoints, and cellular recruitment pathways, may improve the efficacy of existing treatments and reduce resistance mechanisms. Recent studies emphasize the prognostic and predictive value of TIME in breast cancer, suggesting its potential as a biomarker for patient stratification and therapeutic response. Importantly, the interplay between TIME components and molecular subtypes of TNBC underscores the necessity of individualized therapeutic strategies.

### 2.1. Immune Cells in TIME

TIME is a complex network of immune cells, stromal cells, and signaling molecules that interact dynamically with tumor cells. The TIME is composed of various immune cell subtypes, including cytotoxic T lymphocytes (CTLs) [17], TAMs [18,19], tumor-associated neutrophils (TANs) [20], myeloid-derived suppressor cells (MDSCs) [21], T cells (cytotoxic and regulatory) [22,23,24], and mesenchymal stem cells (MSCs) [25]. These cells are recruited and activated by chemokines such as CCL2, which plays a pivotal role in shaping the immune landscape of the tumor [16]. Stromal components, including cancer-associated fibroblasts and cancer-associated adipocytes, contribute to the immunosuppressive and pro-tumorigenic nature of the TIME.

TAMs further shape the TIME, existing in a spectrum from tumoricidal (M1) to immunosuppressive (M2) states, influenced by factors like GM-CSF and IL-10 [26,27,28]. Yolk-sac-derived TAMs (YS-TAMs) are notably immunosuppressive early in tumor development, while monocyte-derived TAMs accumulate as tumors progress, amplifying immunosuppression [29]. Dendritic cells (DCs), particularly CD103+ subsets, are essential for priming antitumor CD8^+^ T cells and are correlated with favorable prognoses and enhanced immune checkpoint blockade (ICB) responses, although their populations decline as tumors advance, compromising T cell activation [12,30,31,32,33,34].

Regulatory T cells (Tregs) suppress immune responses by inhibiting effector T cells and fostering tolerance, and their presence within tertiary lymphoid structures (TLSs) can influence therapeutic outcomes [35,36]. Neutrophils, meanwhile, exhibit both anti-tumor [37,38] and prometastatic [39,40,41,42,43,44] functions—some inhibit metastatic seeding, while others promote metastasis by secreting leukotrienes and dampening immunity [37]. Classical monocytes, drawn by tumor-derived chemokines like CCL2, differentiate into TAMs and support immunosuppression [45], whereas nonclassical monocytes possess antimetastatic properties [46].

### 2.2. Impact of TIME Composition on Tumor Response to Therapy

The TIME can be broadly classified into three categories based on immune cell infiltration and activation levels: (1) Infiltrated-Excluded (I-E) TIME, where immune cells are restricted to the tumor periphery with limited entry into the core, resulting in “cold” tumors that display poor immunogenicity and limited responsiveness to immune checkpoint blockade (ICB); (2) Infiltrated-Inflamed (I-I) TIME, which characterizes “hot” tumors, marked by abundant activated CTLs and expression of immune checkpoint molecules such as PD-L1, leading to more robust responses to ICB; and (3) TLS-TIME, defined by the presence of TLS, which serve as sites for immune activation and recruitment. TLSs composed of various immune cells, including Tregs, B cells, and dendritic cells, are frequently associated with stronger antitumor immunity and improved therapeutic outcomes [12].

In I-I TIME, the presence of high CTL infiltration and immune-activating molecules such as PD-1 and IFN-γ typically results in favorable responses to ICB therapies. For example, MSI-H colorectal cancers, which are enriched with neoepitopes and CTLs, often demonstrate marked sensitivity to anti-PD-1 treatment [12]. Conversely, I-E TIME, where immune cells primarily confined to the tumor periphery, tend to be immunologically “cold” and less responsive to ICB due to immune ignorance or suppression [12,47].

The presence of TLSs within the TIME correlates with improved responsiveness to immunotherapies by promoting recruitment and activation of adaptive immune effectors [12,35]. Furthermore, the tumor’s mutational landscape is pivotal in shaping immune activity: tumors with high mutational burdens, such as MSI-H malignancies, generate abundant neoepitopes that facilitate CTL infiltration and activation, enhancing susceptibility to ICB [12]. In contrast, tumors with low mutational burdens or antigenically “cold” profiles exhibit poor immune infiltration and limited therapeutic responsiveness [12].

Additionally, the spatial distribution of immune cells is critical; when CTLs are sequestered in fibrotic nests or localized at tumor margins, their capacity to mediate anti-tumor effects is substantially hindered, consequently reducing the efficacy of therapy [12]. A further layer of complexity is introduced by the progressive depletion of essential immune activators, such as CD103+ dendritic cells, which are crucial for priming antitumor CD8^+^ T cell responses. The loss of these key cells contributes to diminished immune competence and the attenuated success of ICB-based interventions [30,31,32,33,34].

A comprehensive understanding of both the composition and dynamic behavior of the TIME is imperative for accurate prognostication and the customization of therapeutic approaches. Strategies include, but are not limited to, enhancing CTL infiltration and activation, targeting immunosuppressive elements such as TAMs and Tregs, promoting the establishment of TLSs, and integrating ICB with therapies directed at normalizing the TIME, such as cytokine modulation or metabolic interventions. By systematically addressing the unique attributes of the TIME, therapeutic regimens can be refined to optimize patient outcomes and improve survival rates.

### 2.3. Interplay Between TIME Components and Molecular Subtypes of TNBC

The interplay between the TIME components and molecular subtypes of TNBC highlights the complexity of immune–tumor interactions. It plays a significant role in determining both tumor progression and responsiveness to immunotherapy. The distinct molecular subtypes of TNBC exhibit unique immune landscapes, characterized by varying compositions of immune cells, cytokine profiles, and immune regulatory signals. Understanding these TIME features is critical to developing effective, tailored immunotherapeutic strategies.

Basal-like TNBC typically presents an inflamed or “hot” TIME. Key features include a high tumor mutational burden (TMB) and neoantigen load, which stimulate the robust infiltration of CTLs and the upregulation of PD-L1 on both tumor and immune cells. Spatial profiling studies in TNBC identified a “fully inflamed” TIME subtype marked by tumor-core CD8^+^ T cells, granzyme B positivity, type I IFN signatures, and MHC I expression—hallmarks of immune activation and checkpoint responsiveness [48].

Mesenchymal TNBC is characterized by an immunosuppressive, “immune-excluded” TIME, driven by epithelial-to-mesenchymal transition (EMT), TGF β signaling, and stromal fibrosis. Spatial TIME classification labels tumors as margin-restricted (MR) or immune-desert (ID), describing low CD8^+^ T-cell infiltration, high fibroblast content, TGF-β-dependent stromal activation, B7-H4 expression, and the absence of PD-L1/IDO1, indicative of active immune exclusion. Transcriptomic analyses further associate mesenchymal TNBCs with suppressive checkpoints such as B7 H3 and B7 H4, reinforcing their low immunoreactivity [49].

Immunomodulatory TNBC features an actively inflamed TIME enriched with CTLs, dendritic cells, B cells, and tertiary lymphoid structures (TLSs). Gene-expression analyses indicate the upregulation of co-stimulatory and inhibitory receptors (e.g., OX40, ICOS, PD1, CTLA-4) in this subtype, highlighting a dynamic immune microenvironment poised for activation but still modulated by immune checkpoints. Population-level profiling correlates this “immune-inflamed” TIME cluster strongly with the immunomodulatory molecular subtype, suggesting heightened sensitivity to checkpoint blockade [50].

In contrast, the luminal androgen receptor (LAR) subtype is often relegated to an “immune-desert” or “stroma-restricted” TIME, marked by low immune infiltration, minimal checkpoint expression, and sparse antigen-presentation machinery. Spatial TIME signatures align LAR tumors with fibrotic, CD8-low profiles and low expression of activation markers, underscoring the absence of an adaptive immune presence [48]. This “cold” TIME indicates innate resistance to immunotherapy and prioritizes strategies that induce immune recruitment and activation.

Distinct TIME phenotypes in TNBC subtypes highlight key differences in immune activity and therapy response. Basal-like and immunomodulatory subtypes show active anti-tumor immunity, while mesenchymal and LAR subtypes exhibit immune exclusion or desertion. These variations emphasize the need for subtype-specific treatments such as the use of polyphenols, which have been described to modulate the TIME by reducing immunosuppressive cytokines, reprogramming macrophages, enhancing antigen presentation, and boosting T cell recruitment. Integrating these agents with immunotherapy may help overcome resistance by reconditioning immune-silent or excluded environments with minimal toxicity.

### 2.4. The Effects of the Host Gut Microbiome on the TIME

The presence of bacteria within tumors has been documented for decades. A landmark study by Nejman et al. (2020) validated the presence of intertumoral bacteria across a substantial cohort comprising 1010 tumor samples and 516 matched adjacent normal tissues [51]. Their comprehensive analysis across various solid tumor types—including breast cancer—revealed consistent detection of bacterial components such as lipopolysaccharide (LPS) and 16S rRNA. Notably, breast tumors exhibited a higher microbial diversity, averaging 16.4 distinct bacterial species, in contrast to fewer than nine species identified in other tumor types [52]. These findings underscore the microbiome’s potential to modulate the TIME and suggest tumor-specific microbial signatures that may influence immune responses and therapeutic outcomes.

The heterogeneity of breast cancer has been associated with microbiota-induced changes in metabolic pathways, where modification in the composition of the breast microbiome and the gut can promote the progression of breast cancer [53,54]. A different breast microbiome was described in women with malignant disease, where invasive breast cancer showed an enhanced abundance of Fusobacterium, Atopobium, Hydrogenophaga, Gluconacetobacter, and Lactobacillus [55].

Studies have shown that chemotherapy can modify the breast tumor microbiome, leading to changes in specific microbes, suggesting that the presence of tumor-specific bacteria may be associated with cancer recurrence [56]. The use of neoadjuvant chemotherapy treatments, including the combination of anthracycline, alkylating agents, and taxanes, has been described to shrink the breast tumor before surgical procedures [57]. Studies indicated that neoadjuvant chemotherapy may lead to an increased level of Pseudomonas and diminished Prevotella in breast tumors. The abundance of Brevundimonas and Staphylococcus was described in primary tumors from cancer patients who developed metastasis [56]. Pseudomonas aeruginosa was found in 20% of the normal surrounding mammary tissues and 56% of primary breast tumors, where Pseudomonas aeruginosa modulated cancer cell proliferation and doxorubicin-mediated cell death [56].

In TNBC, Wang et al. confirmed that Clostridiales and related gut metabolite trimethylamine N-oxide (TMAO) were prevalent, with plasma TMAO levels associated with a better response to PD-1 blockade. The TMAO was shown to activate endoplasmic reticulum stress kinase, leading to pyroptosis in cancer cells and enhancing anti-cancer immunity via CD8^+^ T cells, as demonstrated in in vivo assays [58]. While breast cancer cells are not in direct contact with gut bacteria, bacterial metabolites produced by the gut might enter circulation and be transported to the breast tissue [59]. Therefore, these studies demonstrate the importance of microbiomes in modulating TIME and their influence on TNBC progression and therapeutics.

## 3. Polyphenol Classification

Polyphenols are a diverse class of compounds found abundantly in plants. They are characterized by multiple phenol units per molecule, often accompanied by one or more hydroxyl groups [15,60]. Their classification encompasses both flavonoids and non-flavonoids, which are frequently bound to sugars and organic acids, contributing to their varied bioactivity. Polyphenols constitute a broad array of plant-derived antioxidants, with thousands of distinct molecules identified in higher plants. These compounds are widely found in various dietary sources. Their chemical diversity allows multiple functionalities to be widespread across plant products, while others are specific to foods [10].

### 3.1. Dietary Sources of Polyphenols

**Figure 1:**
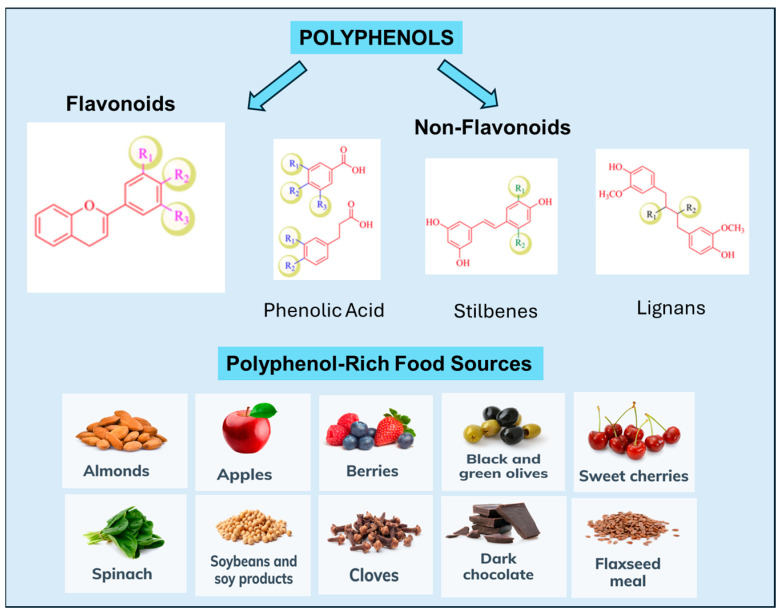
Polyphenol classification and their prevalence in food.

Polyphenols are widely found in various foods, including fruits, vegetables, cereals, seeds, nuts, chocolate, and beverages such as tea, coffee, and wine [14,61]. While thousands of polyphenolic molecules have been identified in plants, several hundred are commonly found in edible plants. Different foods contain specific polyphenols; fruits and beverages like tea and red wine are significant sources [62]. Specific polyphenols, such as quercetin, exhibit wide distribution across plant products, while others, like flavonones and isoflavones, are characteristic of particular foods [62]. Over recent decades, mounting evidence from meta-analyses and observational studies has highlighted the potential of dietary polyphenols in chemoprevention and chemosensitization, particularly in breast cancer [63,64,65,66]. Dietary patterns rich in polyphenols, such as the Mediterranean diet, have been associated with protective effects against breast cancer, particularly in postmenopausal women and TNBC [19,23,24,25] (Figure 1).

### 3.2. Metabolism and Bioavailability of Polyphenols in the Human Body

Estimating daily polyphenol intake is challenging due to several factors, including gender, dietary patterns, and geographic regions. Mediterranean diets are often associated with higher polyphenol intake than Western diets [29]. Typical intake ranges are estimated to be between 0.4 and 1.5 g per individual [36]. However, the health benefits of polyphenols are intricately tied to their bioaccessibility, bioavailability, and subsequent bioactivity within the body [67]. Polyphenol metabolism involves a complex interplay of absorption, transformation, and distribution within the body. Polyphenols undergo extensive metabolism upon ingestion, with their bioavailability influenced by chemical structure, solubility, and membrane permeability. Most polyphenols are present in food as esters, glycosides, or polymers, requiring hydrolysis by intestinal enzymes or colonic microflora for absorption [62]. Subsequently, they undergo phase II metabolism, forming various conjugated metabolites, including sulfates, glucuronides, and methyl derivatives, which may exhibit distinct biological activities [62,63,68,69].

### 3.3. Plasma Transport and Tissue Uptake

Once absorbed, polyphenol metabolites are often bound to plasma proteins, primarily albumin, with varying affinities based on their chemical properties [62]. Plasma concentrations vary depending on the polyphenol and its food source, with cellular uptake proportional to the unbound concentration of metabolites [62]. While tissue uptake patterns remain under exploration, some evidence suggests that certain polyphenols accumulate in specific organs, potentially influencing their biological effects. Studies have demonstrated the presence of polyphenols and their metabolites in breast tissue, albeit at lower concentrations than in plasma [62,70]. At the same time, distribution in breast tissue mirrors plasma concentrations, and certain polyphenols, like curcumin, present challenges due to poor bioavailability [63,71]. Strategies to enhance the bioavailability and stability of polyphenols are being actively explored to maximize their therapeutic potential.

## 4. Molecular Mechanisms of Polyphenols in Modulating TIME in TNBC

Studying the biological impact of polyphenols on TNBC-derived TIME is highly important because of the aggressive nature of this cancer subtype and its limited treatment options. Exploring how dietary compounds like polyphenols interact with TIME could uncover new pathways for treatment. Understanding these interactions may lead to the development of more effective, targeted therapies for TNBC.

Polyphenols exert diverse effects on the TIME through their antioxidant and anti-inflammatory properties, which help counteract immunosuppressive mechanisms and thereby enhance immune surveillance and therapeutic efficacy. Additionally, polyphenols have been shown to regulate angiogenesis, oxidative stress, and immune checkpoint pathways, making them promising candidates for complementary cancer therapies. Dietary flavonoids have been shown to suppress the excessive production of inflammatory mediators such as tumor necrosis factor alpha (TNF-*α*), interleukin-6 (IL-6), mitogen-activated protein kinase (MAPK), nuclear factor kappa-light-chain-enhancer of activated B cells (NF-κB), and inducible nitric oxide synthase iNOS [72].

Polyphenols have gained significant attention for their potential to combat TNBC, primarily through the modulation of inflammatory pathways. Specifically, polyphenols such as curcumin, epigallocatechin gallate (EGCG), resveratrol, cocoa polyphenols, pomegranate polyphenols, and cranberry extract have demonstrated anti-inflammatory properties by targeting key signaling molecules involved in inflammation, including NF-κB, phosphoinositide 3-kinase/protein kinase B (PI3K/Akt), mitogen-activated protein kinase/extracellular signal-related kinase (MAPK/ERK), and signal transducer and activator of transcription 3 STAT3 [73]. The ability of polyphenols to inhibit or block the activity of NF-κB [74,75], cyclooxygenase (COX-2) [75,76,77], and lipoxygenase (LOX) [36,37] is a key contributor to their anti-inflammatory capacity.

The modulatory effect of polyphenols in the inflammatory milieu within the TNBC TIME is remarkable, as the TNBC TIME presents a complex landscape, with the NF-κB signaling pathway playing a significant role in tumor progression and resistance to therapy. Activation of NF-κB, often observed in TNBC, fosters a pro-tumorigenic microenvironment, fueling metastasis and immune evasion [9,10,11,12]. Key players, such as CCL2, also known as monocyte chemoattractant protein-1 (MCP-1), further exacerbate this inflammatory cascade, correlating with a poor prognosis and heightened aggressiveness in TNBC. This promotes tumor growth, invasion, and metastasis by enhancing the recruitment of pro-tumorigenic immune cells to the tumor microenvironment [13,14,15,16]. CCL2 is a pivotal chemokine in inflammation and immune cell recruitment, playing a crucial role in the progression of TNBC. Produced by both tumor and stromal cells within the tumor microenvironment (TME), CCL2 acts as a potent chemoattractant for monocytes and macrophages [17,18,19,20]. Through its interaction with the NF-κB pathway, CCL2 facilitates the recruitment of monocytes and macrophages to the tumor site, promoting tumor growth, invasion, and metastasis [4,21,22,23]. Furthermore, TNBC patients with elevated CCL2 expression tend to have poor prognosis due to the stimulation of immunosuppressive cell recruitment and the activation of NF-κB, which amplifies the inflammatory response [16], creating a pro-tumorigenic milieu conducive to TNBC progression. Studies have shown that human inflammatory breast cancer cell lines expressing elevated levels of CCL2 induce tumors enriched in macrophages [24]. Conversely, CCL2 knockdown significantly reduces macrophage densities, tumor proliferation, skin erythema, and metastasis [24], highlighting the critical role of CCL2 in macrophage expansion and, consequently, tumor growth.

**Figure 2:**
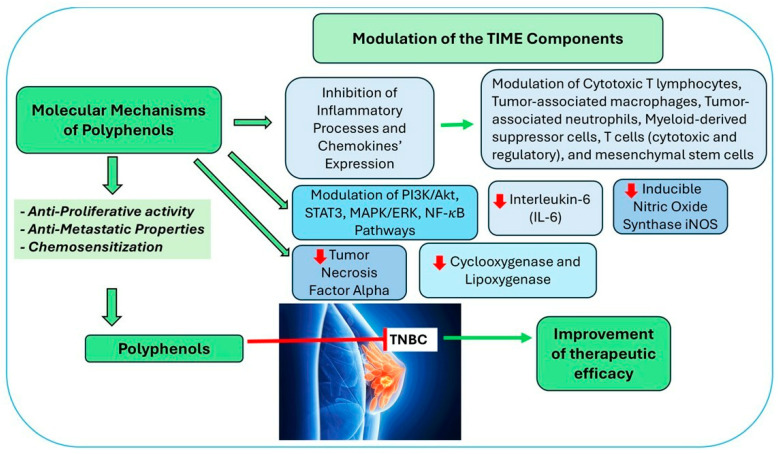
Molecular mechanisms of Polyphenols on the TIME of TNBC. The figure depicts the effect of polyphenols on the expression of several proteins and on the modulation of signaling pathways, ultimately leading to improvement in therapeutic efficacy. Red arrows indicate decreased activation of signaling pathways or reduced expression of proteins.

TIME also plays a crucial role in cancer progression and response to immunotherapy. Over the past decade, cancer treatment has undergone a significant transformation, with antibody-based immunotherapies targeting immune checkpoint inhibitors, such as programmed cell death protein 1 (PD-1) and its ligand (PD-L1), emerging as a significant therapeutic approach for many breast cancer patients [7,25]. The mechanisms through which CCL2 shapes the TIME are tightly regulated by tumor cells, tumor-infiltrating immune cells, and the tumor stroma [20]. In addition to NF-κB, CCL2 expression is enhanced through activating mTOR, a downstream effector of the PI3K/AKT signaling pathway, which promotes tumor-associated macrophage (TAM) recruitment [26]. Notably, blocking the CCL2 receptor, CCR2, has been shown to improve the therapeutic efficacy of PD-1 inhibitors in cancer treatment, suggesting that a combination of anti-PD-1/PD-L1 and CCR2-targeted therapies may provide superior outcomes compared to either treatment alone [27,28] (Figure 2).

### 4.1. The Interaction of Polyphenols and the Microbiome

Polyphenols were described to act in the gut microbiota by inducing the growth of beneficial microbes and slowing down the growth of pathogenic microbiota, such as Lactobacillus and Bifidobacterium, which are considered beneficial probiotics to human health and can indirectly decrease the number of pathogenic microbial species [78,79]. Moreover, the microbiota may affect polyphenols, helping to increase their bioavailability. Polyphenols with high molecular weight can be metabolized by the intestinal microbiota into more bioactive metabolites, improving their bioavailability. Many studies have described that 90% of polyphenols that are ingested become bioavailable products through the action of the gut microbiota [78,79]. Therefore, these interactions could alter human metabolism and diminish cardiometabolic risk, although studies are still scarce and have several limitations [80].

### 4.2. Bidirectional Interactions Between Polyphenols and the Gut Microbiome

Polyphenols have been shown to stimulate the proliferation of beneficial gut microbes such as *Lactobacillus* and *Bifidobacterium*, both of which are recognized as health-promoting probiotics capable of suppressing pathogenic microbial populations [78,79]. High molecular weight polyphenols are particularly noteworthy, as they are metabolized by intestinal microbiota into more bioactive derivatives, significantly enhancing their bioavailability. Notably, approximately 90% of ingested polyphenols transform into bioavailable forms through microbiota-mediated metabolism [78,79].

These reciprocal interactions underscore a dynamic relationship: polyphenols can modulate the gut microbiota by fostering beneficial bacterial growth and suppressing pathogenic species, while the microbiota, in turn, facilitates the conversion of polyphenols into bioactive compounds. This synergy holds potential for influencing host metabolic pathways and mitigating cardiometabolic risk. Despite promising findings, current evidence remains limited, and further investigations are warranted to elucidate the clinical significance of microbiome–polyphenol interactions fully [80].

### 4.3. Curcumin

Curcumin, a polyphenol derived from turmeric, has been shown to modulate the TIME by regulating the composition and function of immune cells, thereby enhancing anti-tumor immunity. Bhattacharyya et al. demonstrated that curcumin inhibits tumor progression by affecting the biological behavior of tumor cells and reshaping the TIME to favor tumor eradication [81]. In the immunosuppressive TIME, tumor-specific antigens and the influence of inhibitory cytokines lead to apoptosis of inactivated CD4^+^ and CD8^+^ T cells and cytotoxic T lymphocytes, a process that curcumin has been shown to reverse effectively [82]. Curcumin has also been shown to enhance T-cell resistance to apoptosis by neutralizing oxidative stress in tumor cells, restoring NF-κB activity, and reactivating the TNF-α signaling pathway [83]. A clinical trial involving colon cancer patients conducted by Xu et al. demonstrated that curcumin inhibited FOXP3 gene transcription and expression, facilitating the conversion of FOXP3+ T regulatory cells into Th1 cells, while also blocking T regulatory cell-induced IFN-γ secretion from CD4^+^ T cells [84]. Similarly, curcumin has been found to inhibit T regulatory cell function in lung cancer [85]. Curcumin has also been shown to modulate cytokine function by both promoting and suppressing specific cytokines, thereby remodeling the tumor immunosuppressive microenvironment [86]. In TNBC, curcumin counteracts the tumor-promoting effects of IL-6, which is known to mediate tumor immune evasion and resistance to vaccine therapy [87]. Moreover, curcumin influences adaptive and innate immunity by counteracting tumor-derived exosomes that suppress natural killer cell activation via the IL-2-dependent Janus kinase/signal transducers and activators of transcription (JAK3/STAT5) pathway [88]. Additionally, curcumin enhances NK cell cytotoxicity by increasing the expression of CD16+ and CD56dim on NK-92 cells [89].

Concerning breast cancer, the effect of an antitumor compound containing resveratrol, curcumin, and quercetin on the immunosuppression of the TIME of breast tumor-bearing mice has recently been studied. Li et al. designed an anti-tumor compound, RCQ (a blend of resveratrol, curcumin, and quercetin), which exhibited no side effects and showed anti-proliferative effects on tumor growth and development. They demonstrated that RCQ remodeled the TIME by increasing T cell recruitment while reducing the numbers of neutrophils and macrophages [90]. RCQ also suppressed tumor-infiltrating lymphocytes from becoming immunosuppressive populations, converting CD4^+^ T cells to Th2 cells, TANs to N2 cells, and TAMs to M2 cells [90]. In vitro, RCQ significantly increased reactive oxygen species, reduced mitochondrial membrane potentials in cancer cells, and modulated pro-apoptotic B-cell lymphoma-2 (Bcl-2) family members, thus enhancing reactive oxygen species (ROS)-induced apoptosis in tumor cells and mitigating immunosuppression to promote anti-tumor effects [90].

In the TIME, a large number of inactivated CD4^+^ and CD8^+^ T cells and CTLs undergo apoptosis because of the absence of effective presentation of tumor-specific antigens and the influence of several cytokines with inhibitory effects [82]; a process that was shown to be reversed by curcumin. Through tumor development, cancer cells can induce thymus atrophy, reduce the production of mature T cells, and enable escape from the adaptive immune response. By the interference with the production of NF-κB in T cells, T cells become more susceptible to TNF-α-mediated apoptosis, which is the way that tumor cells induce apoptosis of T cells [83]. Curcumin was shown to neutralize the oxidative stress of tumor cells, restore NF-κB activity, and reactivate the TNF-α signaling pathway, thus causing the enhancement of T cells’ ability to resist apoptosis [83]. Curcumin’s effect on T cells highlights the importance of this polyphenolic compound, since the literature describes that the mechanisms of tumor cell immune escape are associated with loss of effector and memory T cell subsets, secretion of type II cytokines, and increased proliferation of Tregs [83].

Curcumin has also been described as interacting with microbiomes, where it goes through biotransformation (phase I and II) in the liver and in the cells of the intestine. Through the action of enzymes, curcumin is reduced to dihydrocurcumin, tetrahydrocurcumin, hexahydrocurcumin, and octahydrocurcumin [91]. The metabolites will then be converted into curcumin glucuronide or they will be converted into curcumin sulfate, confirming that the transformation of curcumin requires several enzymes that are produced by the gut microbiota [92]. Furthermore, the literature shows that more intestinal microflora can produce several metabolites through a variety of processes, including acetylation, demethylation, reduction, and hydroxylation. Studies also show that once these metabolites are produced, they have anti-inflammatory and antioxidant properties, showing therapeutic effects [93].

### 4.4. Gossypol

Gossypol, a polyphenol compound derived from the seeds of cotton plants (*Gossypium hirsutum* L.), has garnered significant attention in recent years due to its promising medicinal properties. Numerous studies have explored its potential as an anticancer agent, particularly in various human cancers such as colon [94], prostate [95], glioma [96], adrenal [97], leukemia [98], and breast cancer, including TNBC [99,100,101,102,103].

Breast cancer studies indicated that gossypol presents anticancer activity on MCF7 cells, significantly decreasing cell growth after 24 h [104]. Xiong et al. used MCF-7, MDA-MB-231, MDA-MB-468, ZR-75-1, and T47D cells, showing that MDM2 and VEGF, responsible for tumor progression, were inhibited by gossypol in a time- and dose-dependent manner. In addition, gossypol induced apoptosis in all breast cancer cell lines. Authors also developed an in vivo study using mice xenograft models (MCF-7 and MDA-MB-468) treated intraperitoneally with gossypol (10 mg/kg/day) and observed inhibition of tumor growth in both types of xenograft models after 4 weeks [105].

A study conducted by Morelos-Garnica et al. showcased the behavior of gossypol as a pan-Bcl-2 family inhibitor. Gossypol exhibited differential sensitivity in breast cancer cell lines, with MDA-MB-231 cells being more responsive than MCF-7 cells, possibly due to overexpression of Bcl-2 protein and slight G-protein coupled estrogen receptor 1 (GPER) expression [101]. Messeha et al. provided further insights into the molecular mechanisms underlying gossypol’s anticancer effects in TNBC. Using TNBC cell lines MDA-MB-231 and MDA-MB-468, gossypol was found to modulate the expression of apoptosis-related genes, significantly increasing the expression of GADD45A, TNFRSF9, and BNIP3 while repressing the mRNA expression of genes such as BIRC5, DAPK1, and TP73 [100].

Furthermore, Messeha et al. explored the potential effect of gossypol on pro-inflammatory cytokines, including IL-8 and CCL2, which had not been previously reported. The study compared the effects of gossypol on MDA-MB-231 (Caucasian) and MDA-MB-468 (African American) TNBC cell lines stimulated with TNF-α, revealing its ability to attenuate the expression of cancer-related cytokines in both cell lines. While it attenuated CCL2 expression in Caucasian cells through the repression of inhibitor of nuclear factor kappa B kinase subunit epsilon (IKBKE), CCL2, and MAPK1 gene expression, it inhibited IL-8 expression in African American cells by downregulating IL-8, MAPK1, MAPK3, Coiled-coil domain containing 88A (CCDC88A), STAT3, and PIK3CD gene expression, providing insights into potential racial disparities in TNBC treatment response [99]. These findings suggest that gossypol may offer a promising therapeutic option for TNBC, modulating the TIME by targeting multiple signaling pathways.

### 4.5. Butein

Butein (2′,3,4,4′-tetrahydroxychalcone), a polyphenolic compound found in various plant sources, including Semecarpus anacardium, Dalbergia odorifera, and Rhus verniciflua Stokes, has gained recognition in both traditional herbal medicine formulations and modern research for its diverse pharmacological properties. Widely utilized in herbal medicine formulations and as a food additive in various Asian countries, butein exhibits multiple beneficial properties, including anti-inflammatory, antioxidant, and antimicrobial effects [106,107,108]. Of particular interest as an anticancer therapeutic agent, it has demonstrated efficacy against breast cancer, where it has been shown to inhibit the growth of ER+ MCF-7 cells and impede the migration and invasion of human epidermal growth factor receptor 2 positive (HER2+) SKBR-3 breast cancer cells by suppressing NF-κB-dependent CXCR4 expression [109]. Additionally, butein has been shown to induce apoptosis in MDA-MB-231 cells through mechanisms involving the generation of reactive oxygen species and the dysregulation of ERK1/2 and p38 MAPK signaling pathways [110].

Derivatives of butein have been investigated for their potential to target DDX3, an RNA helicase implicated in various cellular processes, including cell survival, cell cycle regulation, and apoptosis. Rampogu et al. investigated the anticancer properties of butein derivatives against MCF-7 and MDA-MB-231 cell lines, revealing significant reductions in cell viability and DDX3 protein expression. These compounds induced apoptotic cell death, arrested cell cycle progression at the G2/M phase, and downregulated PI3K/AKT signaling, suggesting their potential as targeted therapies [111]. Similarly, Sulaiman et al. highlighted butein’s cytotoxic effects on lung A549 and MDA-MB-231 cells, showing concentration- and time-dependent decreases in cell viability and induction of apoptotic cell death [112]. Notably, butein inhibited cancer cell migration in MDA-MB-231 cells, suggesting its potential as a therapeutic agent for metastatic breast cancer [112].

Despite the promising anticancer effects of butein, its impact on pro-inflammatory cytokine release in ethnically diverse TNBC cells remains unexplored. Recent investigations by Mendonca et al. have explored the ethnic disparities in response to butein treatment in Caucasian (MDA-MB-231) and African American (MDA-MB-468) TNBC cells. Results revealed differential cytotoxicity (0.78–6.25 µM vs. 0.78 µM, respectively) and antiproliferative effects (12.5 µM vs. 6.25 µM, respectively) of butein between the two cell lines, with greater potency observed in MDA-MB-468 cells [4]. While butein effectively downregulated TNF-α-induced CCL2 expression and inhibited cell proliferation in MDA-MB-231 cells, its effects were less pronounced in African American cells [4]. Furthermore, butein inhibited IKBKE mRNA and protein expression in Caucasian cells [4], highlighting its potential as a modulator of inflammatory signaling pathways implicated in TNBC progression. Yang et al. demonstrated that butein inhibits the proliferation of breast cancer cells via the generation of ROS and the modulation of ERK and p38 activities, showing that pre-treatment with the antioxidant, N-acetyl cysteine (NAC), prevented butein-induced apoptosis [110].

Overall, these findings underscore the multifaceted therapeutic potential of butein in modulating the TIME in breast cancer through its cytotoxic, antimetastatic, and anti-inflammatory properties. Further research is warranted to elucidate the precise mechanisms of action and to explore the clinical utility of this approach in diverse cancer types and patient populations.

### 4.6. Epigallocatechin Gallate

EGCG is a prominent flavonoid abundant in green tea. It constitutes more than half of the polyphenols in this beverage [14]. EGCG has garnered considerable attention in cancer research and is renowned for its antitumoral and antioxidant properties. Recent studies, including murine models, have elucidated EGCG’s ability to inhibit the infiltration of TAMs mediated by exosomes through the transfer of miR-16 [113].

EGCG has been shown to exert profound immunomodulatory and metabolic effects on TIME, influencing the functions of both immune and stromal cells, which is crucial in TNBC, where TIME plays a pivotal role in disease progression. EGCG has also been shown to modulate the TIME by promoting the anti-cancer immune response of cytotoxic lymphocytes and dendritic cells; attenuating the immunosuppressive functions of myeloid-derived suppressor cells; and inhibiting tumor-promoting activities of tumor-associated macrophages, neutrophils, and various stromal cells [114].

TNBC cells have been shown to secrete factors that induce a pro-inflammatory phenotype in human adipose-derived mesenchymal stem cells (hADMSC), contributing to tumor progression [115,116]. A pivotal investigation by Gonzalez Suarez et al. delved into EGCG’s impact on the interplay between tumor-derived extracellular vehicles (EVs) and the pro-inflammatory phenotype induced in hADMSC by TNBC cells. Treatment with TNBC-derived EVs upregulated the expression of cancer-associated adipocyte pro-inflammatory markers, including CXCL8, CCL2, and IL-1β, in hADMSC [117]. Conversely, EVs isolated from EGCG-treated MDA-MB-231 cells exhibited a distinct gene expression profile, characterized by the downregulation of CCL2 and IL-1β, alongside elevated levels of CXCL8 and IL-6 [117]. Furthermore, EGCG-EVs have a profound influence on intracellular signaling pathways within hADMSC [117]. While EVs activate checkpoint kinase 2 (CHK-2), c-Jun, AKT, and glycogen synthase kinase 3 beta (GSK-3β) signaling pathways, EGCG-EVs specifically attenuate the latter two pathways and mitigate serum starvation-induced senescence markers [117]. Moreover, EGCG treatment reduces mitochondrial content within TNBC-derived EVs, suggesting a potential mechanism underlying its modulatory effects on EV-mediated paracrine signaling [117]. This intriguing observation sheds light on EGCG’s ability to alter the cargo composition of EVs, potentially influencing their functional properties in the TIME [117].

A clinical trial demonstrated that 80% of patients with chronic lymphocytic leukemia experienced a reduction in circulating lymphocytes and Tregs following EGCG treatment [118]. Evidence from Saleh et al. further supports EGCG’s significant anti-proliferative and immunosuppressive effects on peripheral blood mononuclear cells isolated from newly diagnosed breast cancer patients or age-matched controls stimulated with mitogen, anti-CD3, and cancer antigen peptides, and a significant reduction in IFN-γ production in vitro [119].

These findings highlight EGCG’s multifaceted regulatory effects on intracellular signaling cascades implicated in cellular senescence and inflammation [117]. They also shed light on the intricate molecular mechanisms underlying EGCG’s anti-inflammatory and antitumoral effects. Further elucidation of EGCG’s molecular mechanisms holds promise for developing targeted strategies to modulate the TIME and enhance therapeutic outcomes in TNBC.

### 4.7. Cardamonin

Cardamonin, another flavonoid (chalcone) richly present in plants, has been described as a promising agent against breast cancer [120,121]. In TNBC cells, cardamonin presented cytotoxicity by apoptosis induction and cell cycle arrest by modulating Bcl-2, Bax, cytochrome C, cleaved caspase-3, and poly (ADP-ribose) polymerase (PARP) [121]. Cardamonin reduced cell invasion and migration and decreased the stability of β-catenin nuclear translocation [121]. Moreover, cardamonin inhibited MDA-MB-231 and MCF-7 cell proliferation by promoting G2/M arrest and apoptosis [120].

More recently, Mendonca et al. described cardamonin’s potential to interact with the TIME and modulate different pathways involved in TNBC progression [122]. They demonstrated that cardamonin reduced the expression of mucin 1 (MUC1) and STAT3 in MDA-MB-231 and MDA-MB-468 cells and downregulated the mRNA expression of JAK1 only in the Caucasian cells. Cardamonin also increased the expression of Nrf2 and inhibited the mRNA and protein expression of PD-L1 in both TNBC cell lines. When cardamonin and TNF-α were combined, there was a decrease in CCL2 protein expression in the MDA-231 and MDA-468 cells [122]. CCL2’s distinct mechanisms for regulating the TIME are controlled by the tumor, tumor-infiltrating immune cells, and the tumor stroma. Because of this, CCL2 overexpression can induce tumor metastasis, invasion, and the development of immune resistance, and in cancer patients, it characterizes a poor prognosis due to immunosuppressive cell subtype accumulation [16]. Cardamonin’s effect on PD-L1 and CCL2 expressions suggests that it can lower PD-L1 levels and reduce tumor resistance over time by inhibiting CCL2. This demonstrates the potential anticancer effects of cardamom through its potent ability to modulate the TIME genes and proteins, indicating that cardamom can be considered a candidate for TNBC immune therapy.

### 4.8. Resveratrol

Resveratrol is a naturally occurring polyphenol with potent antioxidant activity, found in red wine, grapes, and berries. Numerous studies have analyzed the effects of resveratrol on the TIME, where it showed a dose-dependent impact on immune responses, with lower doses enhancing immune activity. In comparison, higher doses suppress T-cell proliferation [123]. In vivo studies by Soto et al. have shown that combining resveratrol with IL-2 resulted in a 61% survival rate in treated mice compared to 15% in the resveratrol-only group, suggesting that the immunomodulatory effects of resveratrol are dose-dependent and that polyphenols in general can elicit strong chemo-supportive effects [124]. Resveratrol has also been shown to decrease mRNA levels of iNOS, IL-8, and TNF-α, mediated by selective binding to KH-type splicing regulatory protein, which post-transcriptionally regulates pro-inflammatory genes [125,126]. By modulating mitochondrial functions, resveratrol can influence the production of ROS and other inflammatory mediators. The reduction in ROS levels and the subsequent decrease in oxidative stress can lead to a reduction in the activation of various pro-inflammatory pathways [125,127,128]. Resveratrol has been shown to downregulate the production of pro-inflammatory cytokines, such as TNF-α, IL-6, and IL-1β, thereby disrupting the signals promoting inflammation within the TIME and hindering tumor growth and progression [129]. Han et al. described that administration of resveratrol may inhibit 4T1 lung metastasis and induce suppression of tumor growth in the lungs of tumor-bearing mice [130]. Resveratrol downregulated PD-1 expression on local CD8^+^/CD4^+^ T cells and promoted M1 macrophage polarization, which may contribute to resveratrol-mediated anti-tumor efficacy [130]. The results provided evidence that resveratrol might be a promising candidate agent for adjuvant therapy in the process of TNBC lung metastasis 

Resveratrol interaction with the microbiome has been described, where resveratrol passes through biotransformation (phase II) in the liver, and intestinal cells, through different processes such as sulfation, glucuronidation, and metabolism. In the intestine, by the action of intestinal bacteria, this compound is converted into resveratrol-3-O-sulfate (R3S), resveratrol-4′-O-sulfate (R4S), and resveratrol disulfates [131]. After that, resveratrol is hydrogenated by bacteria (*Slackia equolifaciens* and *Adlercreutzia equolifaciens)* and transformed into dihydroresveratrol, which is in part absorbed and metabolized into monosulfate dihydroresveratrol or monoglucuronide dihydroresveratrol, which can be excreted in urine [132]. Confirming the involvement of microbiomes in the bioavailability and excretion of resveratrol.

### 4.9. Quercetin

Quercetin, a polyphenolic flavonoid abundant in fruits and vegetables, has emerged as a promising natural compound for modulating the tumor immune microenvironment TIME in TNBC. Due to its aggressive phenotype and lack of targeted therapies, TNBC presents a significant therapeutic challenge. A recent study by Abdel-Latif et al. has highlighted quercetin’s capacity to influence key immune and molecular pathways that underpin tumor progression and immune evasion. A methoxylated quercetin glycoside (MQG) derived from Cleome droserifolia demonstrated selective cytotoxicity against TNBC cells (IC_50_ = 12 µM), suppressing proliferation, invasion, and migration (Abdel-Latif). Mechanistically, MQG modulated a novel ceRNA axis—MALAT-1/TP53/miR-155/miR-146a—leading to the suppression of nitric oxide production via downregulation of NOS2 and NOS3 [133]. Additionally, MQG upregulated NK cell-activating ligands (MICA/B, ULBP2, CD155, ICAM-1) and suppressed immunosuppressive cytokines (TNF-α, IL-10), enhancing NK cell-mediated cytotoxicity and alleviating immune suppression within the TIME [133].

Complementary findings from another study by Liao et al. underscore quercetin’s broader immunomodulatory effects through inhibition of the IL-6/JAK2/STAT3 signaling pathway—a key driver of tumor growth and immune evasion in TNBC. In vitro and in vivo models demonstrated that quercetin reduced tumor cell proliferation, induced apoptosis, and significantly depleted Tregs in tumor-draining lymph nodes. This led to a reduction in IL-10, an increase in TNF-α, and enhanced cytotoxicity of CD8^+^ T cells and NK cells through activation of the Granzyme B/B/Perforin and Caspase-3 pathways (Liao). Notably, quercetin displayed low toxicity in animal models, supporting its potential as a safe adjunct to immunotherapy [134].

Together, these studies highlight quercetin and its derivatives as multi-targeted, low-toxicity agents capable of reprogramming the TIME in TNBC. Through modulation of both tumor-intrinsic and immune-related pathways, quercetin offers a compelling strategy to enhance antitumor immunity and overcome resistance in this challenging breast cancer subtype. Further preclinical and clinical investigations are warranted to validate these findings and explore their utility in combinatorial regimens.

Quercetin and rutin (its derivative) interact with the microbiome by passing through biotransformation (phase I and phase II) and being absorbed by the small intestine. Before intestinal absorption, quercetin is frequently presented as glycosides, while rutin is first deglycosylated into quercetin aglycon (phase I biotransformation) [135]. Then, quercetin aglycon passes through biotransformation (phase II) in the intestinal cells. Parts of the compounds that are not absorbed are then metabolized by the colon microflora. Initially rutin is hydrolyzed to quercetin by the enzyme β-glucosidase, which is derived from intestinal microbiota, and then quercetin is produced by the action of several bacteria (*Eubacterium ramulus*, *Clostridium orbiscindens*, *Eubacterium oxidoreducens*, and *Butyrovibrio* spp.) to produce phenolic compounds with low molecular weight, which can be absorbed [136,137], showing that the microbiome may affect the bioavailability and effectiveness of quercetin.

## 5. Clinical Evidence Supporting the Anticancer Effects of Polyphenols on the TIME in TNBC

While preclinical studies have provided insights into the mechanisms of action of polyphenols against TNBC, clinical evidence supporting their anticancer effects remains limited and inconsistent. Clinical trials evaluating polyphenol supplementation in TNBC patients have yielded mixed results, and epidemiological studies linking dietary polyphenol intake with TNBC risk and outcomes have not consistently demonstrated significant associations. More translational research on polyphenols is crucial for bridging laboratory findings to clinical purposes. Specific emphasis should include the combination of treatments with polyphenols and immune checkpoint inhibitors, such as anti-PD-1/PD-L1, or inhibitors that target JAK/STAT3, PI3K/AKT/mTOR, and NF-κB pathways. Also, the ability of polyphenols to inhibit PD-L1 expression and modulate immune suppressive pathways shows their potential to enhance immunotherapy outcomes [138,139,140], and the development of predictive biomarkers together with the targeting of CCL2 may be extremely important to help with optimizing therapeutic strategies and reducing therapy resistance [141,142,143]. Further, addressing polyphenols’ pharmacokinetic limitations using advanced drug delivery systems (such as nanoparticles, liposomes, and encapsulation) may improve bioavailability and tumor-selective uptake [144,145,146].

Further investigations involving large cohorts are imperative to discern the long-term impact of polyphenols on breast cancer fully. Moreover, conducting preclinical studies remains pivotal in delineating the intricate mechanisms of action of polyphenols, specifically on TNBC [147].

## 6. Conclusions

Breast cancer, particularly TNBC, presents significant challenges in terms of diagnosis, treatment, and disparities in outcomes, especially among underserved populations such as African American women. TNBC presents unique therapeutic challenges due to its aggressive nature and lack of responsiveness to standard treatments. The TNBC TIME contributes to tumor progression and resistance to therapy. Consequently, there is an urgent need for novel therapeutic approaches to address these challenges.

**Figure 3:**
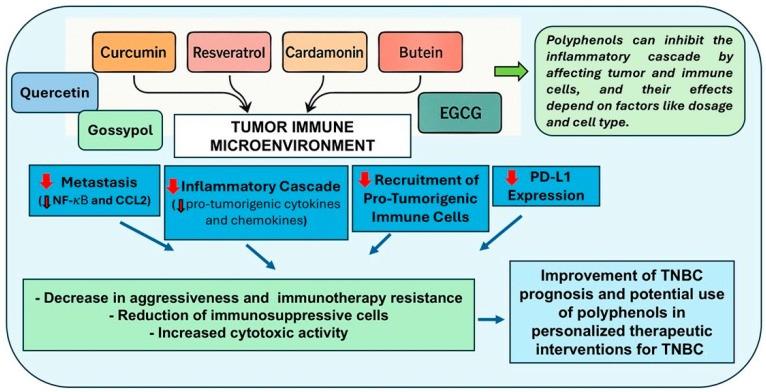
Summary of polyphenols’ effects in the TIME of TNBC. The figure describes the effects of curcumin, resveratrol, cardamonin, butein, quercetin, gossypol, and EGCG on TNBC, and their potential use in personalized therapeutic treatment to improve prognosis. Red arrows indicate decreased activation of signaling pathways or reduced expression of proteins.

Polyphenols, a diverse class of plant-derived compounds renowned for their anti-inflammatory, antioxidant, and anticancer properties, have emerged as promising candidates for TNBC therapy. Through their ability to modulate the TIME by inhibiting inflammatory cascades, PD-L1 and CCL2 expression, reducing immune-suppressive cells and metastasis, and inhibiting the recruitment of pro-tumorigenic immune cells, as well as other cancer development processes, polyphenols offer potential avenues for combating TNBC aggressiveness and improving patient outcomes. Compounds such as curcumin, gossypol, butein, EGCG, cardamonin, quercetin, and resveratrol have demonstrated efficacy in modulating the TIME, thus highlighting their potential in mitigating TNBC tumor progression, and targeting key molecular pathways implicated in TNBC pathogenesis (Figure 3). Moreover, understanding the relationship between the specific microbiota responsible for the metabolism of polyphenols and their metabolized forms is fundamental to investigating the successive health effects of dietary polyphenols. The personalized determination of a patient’s microbiome composition may represent a prospective diagnostic and prognostic instrument, and restoration of balance in microbial community may improve therapeutic efficacy and patient outcomes.

While preclinical studies have provided valuable insights into the mechanisms of action of polyphenols against TNBC, clinical evidence supporting their anticancer effects needs to be more extensive and consistent. Understanding the complex interplay between polyphenols and TIME, particularly the immune response and stromal interactions, is crucial for optimizing therapeutic strategies.

Future research endeavors should focus on elucidating the precise mechanisms of action of polyphenols, optimizing their bioavailability and delivery systems, and conducting rigorous clinical trials to validate their efficacy in diverse patient populations. By harnessing the therapeutic potential of polyphenols, we can strive towards more effective and personalized treatment approaches for TNBC, improving outcomes and reducing disparities in breast cancer outcomes and care.
